# Mental health of people living with dementia in care homes during COVID-19 pandemic

**DOI:** 10.1017/S1041610220001088

**Published:** 2020-06-03

**Authors:** Latha Velayudhan, Dag Aarsland, Clive Ballard

**Affiliations:** 1 Department of Old Age Psychiatry, Institute of Psychiatry, Psychology and Neuroscience, King’s College London, London SE5 8AF, UK; 2 Stavanger University Hospital, Centre for Age-Related Research, 4000 Stavanger, Norway; 3 The University of Exeter Medical School, St Luke’s Campus, Magdalen Road, Exeter EX1 2LU, UK

The COVID-19 pandemic has been causing significant challenges across all areas of society. By virtue of prevalent frailty and multimorbidity, care home residents remain vulnerable to COVID-19 until effective vaccines are developed (Gordon *et al.*, 2020). The current health crisis and the enforced isolation can have huge impact to the mental health of this vulnerable individuals, the majority of whom have dementia and are also at significantly increased risk of severe COVID-19.

At least two-thirds of care home residents and probably more than 80% have dementia (Prince, 2014). Ninety-eight percent of people living with dementia (PlwD) experience neuropsychiatric symptoms over the course of their disease (Vik-Mo *et al.*, 2020). With the ongoing COVID-19 viral pandemic, older people, especially PlwD and care-dependent, may become more anxious, agitated, lonely, and depressed during the outbreak or while in isolation, with huge impact on quality of life, care burden, and use of resources. The increased morbidity and mortality in elderly and care home residents will further affect mental well-being with fear of illness and death in the residents. PlwD will be unable to understand the information on social distancing or to practice it. They will have confusion and distress around relatives not visiting. Limited social interaction and activities with fellow residents, families, and staff due to social distancing can also lead to boredom, inactivity, and sedentary behaviour in residents, further leading to loneliness. Both loneliness and social isolation have been linked to poor mental health (e.g., depression, hopelessness, and cognitive impairment), as well as worse physical health (e.g., worse motor function, cardiovascular health, disrupted sleep, frailty), and higher mortality (Leigh-Hunt *et al.*, 2017; Santini *et al.*, 2020). Imposed isolation can also result in sedentary behaviour which is critical in the prevention of physical, psychological, and social health problems (Maher and Conroy, 2017). It must also be frightening to PlwD when people approach them using personal protective equipment for interpersonal care (care home staff) or by health professionals. With current pandemic, there is a risk for increased use of antipsychotics, hypnotics, and other sedatives in residents with behaviour problems (such as wandering, agitation, or aggression) to ensure compliance with social distancing and to cope with staff shortage. Substantial work has been going on to optimise the use of medications especially antipsychotics, as they have harmful side effects in elderly and especially those with PlwD, and its increased use can double death rates and triple the stroke rates, with the potential to undo more than 10 years of significant progress in the rates of use (Kales *et al.*, 2019; Romeo *et al.*, 2019) and we have evidence from Randomised Controlled Trials and big data studies that the reduction in antipsychotics has led to 30% reductions in both mortality and strokes in this vulnerable population (Sultana *et al.*, 2019). There is a serious concern that without proactive training and support in care homes, that the current pandemic will reverse all of this progress and also lead to significant increases in morbidity and mortality that compound the effects of COVID-19.

We have demonstrated that the Well-being and Health for people with Dementia (WHELD) training programme to promote person-centred care and person-centred activities significantly improve quality of life for PlwD (Kales *et al.*, 2019), as well as reducing neuropsychiatric symptoms and reduce the use of antipsychotic drugs, and recently demonstrated that an eLearning version of the programme also significantly improves the well-being of residents (McDermid *et al.*, 2018). In the light of the pandemic, there is urgent need for innovative training interventions and non-pharmacological approaches for neuropsychiatric symptoms in PlwD that are person-centred, easy to deliver, and culturally competent to improve health outcomes within care home settings and avoid unnecessary hospital admissions during these tough periods of social distancing and isolation.
